# Supplementary material for: Ligand sensing enhances bacterial flagellar motor output via stator recruitment
Source: eLife. 2021 Apr 6;10:e62848. doi: 10.7554/eLife.62848 (PMC8062133; doi:10.7554/eLife.62848)
Supplement: Transparent reporting form [file elife-62848-transrepform.docx]

***eLife’s* transparent reporting form**

We encourage authors to provide detailed information *within their submission* to facilitate the interpretation and replication of experiments. Authors can upload supporting documentation to indicate the use of appropriate reporting guidelines for health-related research (see [EQUATOR Network](http://www.equator-network.org/%20)), life science research (see the [BioSharing Information Resource](https://biosharing.org/)), or the [ARRIVE guidelines](http://www.plosbiology.org/article/info:doi/10.1371/journal.pbio.1000412) for reporting work involving animal research. Where applicable, authors should refer to any relevant reporting standards documents in this form.

If you have any questions, please consult our Journal Policies and/or contact us: [editorial@elifesciences.org](mailto:editorial@elifesciences.org).

**Sample-size estimation**

- You should state whether an appropriate sample size was computed when the study was being designed
- You should state the statistical method of sample size computation and any required assumptions
- If no explicit power analysis was used, you should describe how you decided what sample (replicate) size (number) to use

The sample size and the statistical method is described in captions of Figures 3, 4 and supplementary Figures S4, S5, S6 and S7.

Please outline where this information can be found within the submission (e.g., sections or figure legends), or explain why this information doesn’t apply to your submission:

**Replicates**

- You should report how often each experiment was performed
- You should include a definition of biological versus technical replication
- The data obtained should be provided and sufficient information should be provided to indicate the number of independent biological and/or technical replicates
- If you encountered any outliers, you should describe how these were handled
- Criteria for exclusion/inclusion of data should be clearly stated
- High-throughput sequence data should be uploaded before submission, with a private link for reviewers provided (these are available from both GEO and ArrayExpress)

The number of independent experiments stated in the caption of Figures 3, 4 and supplementary Figures S4, S5, S6 and S7 represent biological replicates performed on three or four different days. For each day, a separate batch of cells was grown for experiments.

For membrane potential measurements (Figures 3D, 4A, 4C, 4E), three technical replicates were used to obtain average value for a single day.

For run speed measurement (Figures 3E, 4B, 4D, 4F), at each time point, five to six images were captured to obtain an average value. This constitutes the technical replicates.

Single cell studies for measurement of motor speed and stator intensity were performed on six different days. More than 20 motors were measured for each ligand, which constitute the biological replicates.

No outliers were encountered. No high-throughput sequence data was used.

Please outline where this information can be found within the submission (e.g., sections or figure legends), or explain why this information doesn’t apply to your submission:

**Statistical reporting**

- Statistical analysis methods should be described and justified
- Raw data should be presented in figures whenever informative to do so (typically when N per group is less than 10)
- For each experiment, you should identify the statistical tests used, exact values of N, definitions of center, methods of multiple test correction, and dispersion and precision measures (e.g., mean, median, SD, SEM, confidence intervals; and, for the major substantive results, a measure of effect size (e.g., Pearson's r, Cohen's d)
- Report exact p-values wherever possible alongside the summary statistics and 95% confidence intervals. These should be reported for all key questions and not only when the p-value is less than 0.05.

Please outline where this information can be found within the submission (e.g., sections or figure legends), or explain why this information doesn’t apply to your submission:

The statistical methods and values of N are detailed in the caption of Figures 3, 4, S4, S5, S6 and S7. Measured standard error or deviation is also clearly stated.

---------------------------------------

Figure 3A-C:

---------------------------------------

Of all the cells imaged in the tethered cell experiment, we chose only those
rotating cells which were tethered at their polar ends. For cells, which
paused for significant durations or stopped completely during the experiment were not considered. Hence only those cells which were properly tethered for the duration of the experiment, about 15 or 20 mins, and rotated without pausing (especially during imaging) were considered. Following are the number of cells that satisfied the aforementioned condition and showed increase in fluorescence:

a) Glucose: 21 cells.
b) 2Dg: 22 cells
c) MB: 21 cells.

Some cells (less than 10%) have been excluded, which showed increase in rotation speed but not in intensity. This is attributed to either weak photobleaching or due to proteolytic activity, which cleaves the fluorescent moiety, leaving some population of MotB non-fluorescent. If stators with cleaved moieties bind to the motor, the speed will increase without a change in intensity.

(For large datasets, or papers with a very large number of statistical tests, you may upload a single table file with tests, Ns, etc., with reference to sections in the manuscript.)

**Group allocation**

- Indicate how samples were allocated into experimental groups (in the case of clinical studies, please specify allocation to treatment method); if randomization was used, please also state if restricted randomization was applied
- Indicate if masking was used during group allocation, data collection and/or data analysis

Please outline where this information can be found within the submission (e.g., sections or figure legends), or explain why this information doesn’t apply to your submission:

Not Applicable

**Additional data files (“source data”)**

- We encourage you to upload relevant additional data files, such as numerical data that are represented as a graph in a figure, or as a summary table
- Where provided, these should be in the most useful format, and they can be uploaded as “Source data” files linked to a main figure or table
- Include model definition files including the full list of parameters used
- Include code used for data analysis (e.g., R, MatLab)
- Avoid stating that data files are “available upon request”

Please indicate the figures or tables for which source data files have been provided:

None
